# A New Model Based on 25-Hydroxyvitamin D3 for Predicting Active Crohn's Disease in Chinese Patients

**DOI:** 10.1155/2018/3275025

**Published:** 2018-12-16

**Authors:** Sinan Lin, Ying Wang, Li Li, Peng Chen, Ren Mao, Rui Feng, Yun Qiu, Yao He, Baili Chen, Zhirong Zeng, Minhu Chen, Shenghong Zhang

**Affiliations:** Division of Gastroenterology, The First Affiliated Hospital, Sun Yat-sen University, Guangzhou, China

## Abstract

**Background:**

The association between vitamin D3 and activity of Crohn's disease (CD) is unclear in Chinese patients. In this study, we aimed to evaluate the correlations between serum levels of 25-hydroxyvitamin D3 (25(OH)D3) and disease activity and predict active disease based on vitamin D status.

**Methods:**

Between January 2014 and December 2017, 346 CD patients from the First Affiliated Hospital of Sun Yat-sen University were recruited and categorized into a group with 25(OH)D3 ≤ 20 ng/ml and a group with 25(OH)D3 > 20 ng/ml. The clinical characteristics, medication, and health-care needs were compared between the groups. The correlations among 25(OH)D3 and routine serum biomarkers and disease activity were examined. The predictive efficiency of 25(OH)D3 and other biomarkers for active diseases was also explored using receiver-operating characteristic (ROC) curve analysis. A new predictive model, −(5^∗^25(OH)D3 + 2^∗^Hb) + ESR, and a nomogram were established using Logistic Regression.

**Results:**

Patients with 25(OH)D3 ≤ 20 ng/ml had higher serum levels of C-reactive protein (CRP), erythrocyte sedimentation rate (ESR), and platelets (PLT) and lower levels of hemoglobin (Hb) and albumin (ALB). Serum levels of 25(OH)D3 were inversely correlated with the score of Crohn's Disease Activity Index (CDAI) (*r*_s_ = −0.608). ROC analysis showed a better predictive value of −25(OH)D3 and the new model with areas under curve (AUC) of 0.804 and 0.879, respectively, than those of CRP (0.693) and ESR (0.713) in disease activity. A nomogram for prediction was established with a c-index of 0.882.

**Conclusions:**

Serum levels of 25(OH)D3 negatively correlated with CD activity in Chinese patients. The new model and a nomogram based on 25(OH)D3 showed a better efficiency in predicting disease activity in CD patients but warrants further study.

## 1. Introduction

Crohn's disease (CD) is a chronic inflammatory disorder characterized by manifestations like diarrhea, abdominal distension, pain, and weight loss [1]. Although the mechanism of CD is still not fully understood, it is believed that CD is closely related to the dysfunction of the immune system including innate and adaptive immune responses. Our previous studies have shown that different kinds of cytokines are involved in the pathogenesis and development of IBD including proinflammatory miR-223 in the intestinal barrier [2] and serum interleukin-9 levels predict disease severity as well as the response to infliximab [3]. Furthermore, different immune cells such as monocytes, T cells, and natural killer cells (NK cells) are involved in the development of CD accompanied by interactions of different cytokines including interleukin-6 (IL-6) and tumor necrosis factor-*α* (TNF-*α*) [4]. One of the most important immune regulators is vitamin D [5].

Vitamin D is a fat-soluble vitamin absorbed from dietary or cutaneous routes with multiple functions [6], which can be well measured by the level of serum 25-hydroxyvitamin D3 (25(OH)D3) [7]. As an important regulator, vitamin D is essential in CD inflammation [8], including reduction in CD4^+^ T cell proliferation, stimulation of NOD2/CARD15/IBD1 gene expression in monocytes and epithelial cells, reduction of immune responses, and maturation of dendritic cells. Vitamin D also has regulatory effects on cytokines such as reducing production of Toll-like receptor-induced cytokines (IL-12/IL-23p40, TNF-*α*, and IL-23) as well as increasing cytokines induced by NOD2 and TLR coactivation (IL-10 and IL-23) [9–13].

The importance of vitamin D in CD has gained attention recently. Data from a prospective cohort study including 72,719 women in the Nurses' Health Study showed that higher predictive levels of 25(OH)D3 significantly reduce the risk of CD but not ulcerative colitis [14]. However, another study from Europe recently demonstrated that vitamin D status was not associated with the risk of CD by the measurement of serum 25(OH)D3 but might still be limited by the relatively small sample size [15]. Although whether vitamin D deficiency is a risk factor for CD is still controversial using vitamin D status before diagnosis, previous studies have reported that the prevalence of vitamin D deficiency is much higher in CD patients than in healthy controls [16]. The correlation between vitamin D status and CD activity was first reported by Harries et al. in a cohort recruiting 40 CD patients [17]. In the last decade, an increasing amount of literature has been published on this topic, but no consensus has been reached. Several studies have demonstrated a negative association between 25(OH)D3 levels [18–20] and CD activity using Crohn's Disease Activity Index (CDAI) or Harvey-Bradshaw index (HBI) while other studies could not find such correlations [21–23].

Despite the increasing concerns on vitamin D in CD, no studies have examined the predictive efficiency of vitamin D for CD activity. Besides, most previous studies were performed on patients in Western countries while only few of them showed characteristics of Chinese patients [24–26]. However, these two studies were restricted by small sample size. Further, uncertainty still exists with respect to the relationship between vitamin D status and CD disease activity in Chinese patients, and there is a need for further research.

Therefore, this study set out to determine the correlation between serum vitamin D levels and disease activity in Chinese people. Furthermore, we used serum 25(OH)D3 for CD activity prediction.

## 2. Materials and Methods

### 2.1. Patients

Between January 2014 and December 2017, patients diagnosed with CD and followed up at the First Affiliated Hospital of Sun Yat-sen University were identified. The diagnosis of CD was based on clinical manifestations, abdominal imaging, and intestinal pathology. A total of 346 patients with definite diagnosis and available records of 25(OH)D3 concentration in serum samples within one month before or after follow-up were included in the study. The data of these patients were extracted retrospectively from their medical records. The exclusion criteria included the absence of records on 25(OH)D3 levels or incomplete data for disease activity (CDAI or HBI). Patients with pregnancy, autoimmune diseases, irritable bowel syndrome, a history of malignancy, and ongoing infection during their first visit were also excluded. Also, no patients with any current treatment of vitamin D were included in this study.

### 2.2. Description of Variables

For these patients, data including gender, age, duration of the disease, previous major intestinal surgery, disease location, behavior, presence of perianal lesions, extraintestinal manifestations, and levels of serum 25(OH)D3 at the first visit were recorded. To determine the severity of disease, systemic inflammation, and nutrient status of patients, we also recorded the score of Crohn's Disease Activity Index (CDAI) or Harvey-Bradshaw index (HBI, when CDAI is unavailable), Simplified Endoscopic Score for Crohn's disease (SESCD, if without previous intestinal surgery), body mass index (BMI), and serum parameters like C-reactive protein (CRP), erythrocyte sedimentation rate (ESR), counts of white blood cells (WBC) and platelets (PLT), hemoglobin (Hb), and albumin (ALB) levels at the first visit. Additionally, we also recorded the time needed for abdominal imaging, IBD-related hospital admission, and IBD-related bowel surgery during follow-up.

Montreal classification was applied to the location and behavior of disease [27]. Extraintestinal manifestations included arthritis, cutaneous lesions, stomatitis, uveitis, and hepatobiliary manifestations.

The serum 25(OH)D3 levels were recorded for determining the status of vitamin D in CD patients. Levels of ≤20 ng/ml denoted vitamin D deficiency, 20–29 ng/ml (20 ng/ml and 30 ng/ml not included) denoted vitamin D insufficiency, and ≥30 ng/ml denoted vitamin D sufficiency. To evaluate the effects of vitamin D deficiency and simplify the interpretation of data, we combined vitamin D insufficiency and sufficiency as the “group with 25(OH)D3 > 20 ng/ml,” while vitamin D deficiency as the “group with 25(OH)D3 ≤ 20 ng/ml.” According to the sun exposure and latitude of South China, we divided the whole year into two periods marking the period with high sun exposure (from April to September) and low sun exposure (from October to March), which might influence the result of serum levels of 25(OH)D3. As for disease activity, CDAI < 150 denoted clinical remission while CDAI ≥ 150 denoted clinical activity. Only when CDAI was not available was HBI applied for the assessment of disease activity, with HBI > 4 denoting disease activity. In the present study, the disease activity of 18 patients was assessed using HBI. Furthermore, SESCD ≤ 2 denoted mucosal healing including complete (SESCD = 0) and incomplete (SESCD = 1 or 2) healing [28].

### 2.3. Data Analysis

IMB SPSS, version 22.0 (IBM Corporation, Armonk, NY), was used for data analysis. Discrete data were reported as frequency and percentage, using a chi-square test or Fisher's exact test for comparison. The continuous variables which were normally distributed were expressed as the mean and standard deviation (SD) while non-normally distributed ones as the median and interquartile range (IQR), using an independent sample *t*-test or Mann–Whitney *U* test for comparison as appropriate. Spearman rank correlation analysis was used for investigating the association between 25(OH)D3 and CDAI and SESCD as well as CRP, ESR, PLT, Hb, and ALB. A chi-square test was used for comparing the percentage of corticosteroid, immunomodulator, infliximab use, and IBD-related bowel surgery within one year after follow-up. The time needed for IBD-related hospital admission, abdominal imaging, and endoscopy in the half, one, and two years after follow-up were compared by Mann–Whitney *U* tests. Logistic Regression was used to establish the new predictive model. The areas under the receiver-operating characteristic (ROC) curves (AUCs) were used to figure out the predictive efficiency of −25(OH)D3, CRP, ESR, and the new model of −(5^∗^25(OH)D3 + 2^∗^Hb) + ESR for active CD. A *Z* test was used for comparing the differences in the AUCs of the ROC between different biomarkers. A nomogram was established based on serum parameters for predicting active disease. All statistical tests were performed 2-tailed with *P* < 0.05 denoting a statistical significance.

### 2.4. Ethical Considerations

The hospital's institutional review board approved the protocol, and every patient in this study provided written informed consent.

## 3. Results

### 3.1. Demographics and Clinical Characteristics

A total of 346 CD patients with serum 25(OH)D3 levels were enrolled in two groups with one group with 25(OH)D3 ≤ 20 ng/ml (*n* = 286) and the other with 25(OH)D3 > 20 ng/ml (*n* = 60). Overall, only 3.2% (*n* = 11) patients had vitamin D sufficiency. The median level of serum 25(OH)D3 was 12.0 ng/ml (IQR: 8.0–17.0 ng/ml). There was no difference in sun exposure between the two groups (*P* = 0.886). Data on demographics and clinical features of both groups are summarized in Table 1. Except for the higher proportion of male patients in the group with 25(OH)D3 > 20 ng/ml (86.7% vs. 68.9%, *P* = 0.005), other clinical characteristics including age, disease duration, location, behavior, perianal lesions, extraintestinal manifestations, previous bowel surgery, and medication were of no difference between the two groups.

The levels of ESR, CRP, and PLT were significantly higher in patients in the group with 25(OH)D3 ≤ 20 ng/ml compared with others (40.0 [IQR: 21.0–62.3] mm/h vs. 28.0 [IQR: 13.5–48.8] mm/h, *P* = 0.004; 20.6 [IQR: 5.5–46.9] mg/dl vs. 11.7 [IQR: 1.9–24.2] mg/dl, *P* = 0.005; 341.5 [IQR: 266.5–413.3] ∗10^9^/l vs. 279.0 [IQR: 224.0–338.8] ∗10^9^/l, *P* < 0.001, respectively) (see Table 2). Furthermore, the levels of Hb and ALB were lower in patients with 25(OH)D3 ≤ 20 ng/ml (110.0 [IQR: 93.8–125.0] g/l vs. 127.0 [IQR: 112.5–139.5] g/l, *P* < 0.001; 34.6 [±5.5] g/l vs. 38.0 [±5.1] g/l, *P* < 0.001, respectively). No significant difference was found in WBC between the two groups.

### 3.2. Correlation of 25(OH)D3 Levels with Disease Activity and Inflammatory Markers

To compare the clinical and endoscopic activity of CD, participants were divided into active disease and clinical remission according to the score of CDAI or HBI. Meanwhile, patients with available endoscopic results (*n* = 282) were categorized into mucosal healing and mucosal activity according to the score of SESCD. The results of these analyses showed that patients with 25(OH)D3 > 20 ng/ml were more likely to be in clinical remission and mucosal healing than patients in another group (12.9% vs. 48.3%, *P* < 0.001; 12.7% vs. 28.9%, *P* = 0.005, respectively). Patients in clinical remission had higher 25(OH)D3 levels than others (19.0 [IQR: 14.0–22.0] ng/ml vs. 10.5 [IQR: 7.0–15.8] ng/ml, *P* < 0.001).

The serum 25(OH)D3 levels were strongly correlated with the CDAI (*r*_s_ = −0.608, *P* < 0.001). For the 18 patients whose disease activity was assessed by HBI, the correlation was similar but weaker than CDAI (*r*_s_ = −0.516, *P* = 0.028), which may probably be due to the small sample size. Correlation between levels of 25(OH)D3 and the SESCD score (*r*_s_ = −0.290), BMI (*r*_s_ = 0.176), ESR (*r*_s_ = −0.205), CRP (*r*_s_ = −0.219), PLT (*r*_s_ = −0.274), Hb (*r*_s_ = 0.370), and ALB (*r*_s_ = 0.351) was weaker but of statistical significance (all *P* < 0.05) (see Table 3). In our study, the CDAI score was poorly correlated with the SESCD score (*r*_s_ = 0.303, *P* < 0.001).

Correlation between ESR and CRP and the CDAI score was also determined in this study. ESR and CRP were positively correlated with the CDAI score (*r*_s_ = 0.391, *P* = 0.001; *r*_*s*_ = 0.392, *P* < 0.001, respectively). The absolute value of the correlation coefficient (*r*_s_) between 25(OH)D3 levels and the CDAI score was significantly higher than that of ESR and CRP (both *P* = 0.002).

### 3.3. Receiver-Operating Characteristic Analysis

**Figure 1:**
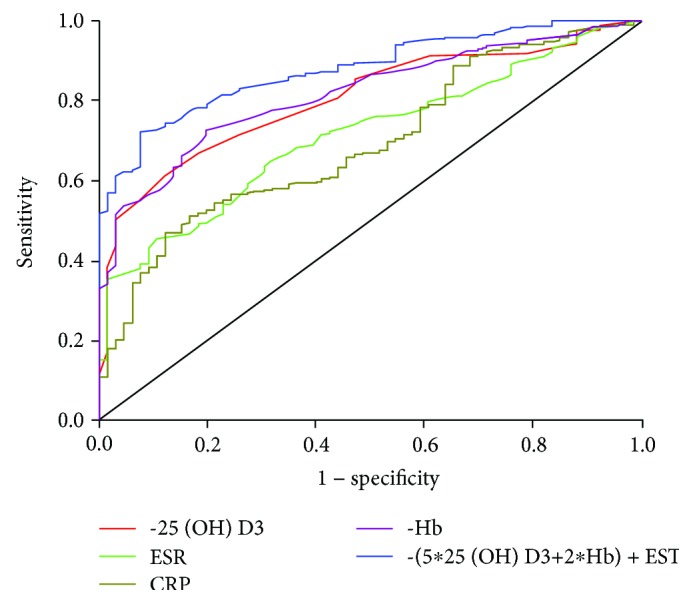
Receiver-operating characteristic curves of various parameters for active disease. The areas under the receiver-operating characteristic curve were 0.804 and 0.816 for −25(OH)D3 and −Hb, respectively, higher than those of CRP (0.693) and ESR (0.713) for distinguishing active from inactive Crohn's disease with *P* < 0.005. The areas under the receiver-operating characteristic curve for −Hb vs. −(5^∗^25(OH)D3 + 2^∗^Hb) + ESR were higher than those of −25(OH)D3 and −Hb with *P* < 0.005.

Receiver-operating characteristic analysis (see Table 4 and Figure 1) showed that −25(OH)D3 had a higher diagnostic accuracy for active disease with an AUC of 0.804 compared to CRP (AUC = 0.693, *P* = 0.0082) and ESR (AUC = 0.713, *P* = 0.0245). The optimal threshold of −25(OH)D3 for active disease was −12.5 ng/ml, with 61.1% sensitivity and 87.9% specificity. There was no difference in AUC between CRP and ESR (*P* = 0.6587).

### 3.4. A New Model Based on 25(OH)D3 and Nomogram

**Figure 2:**
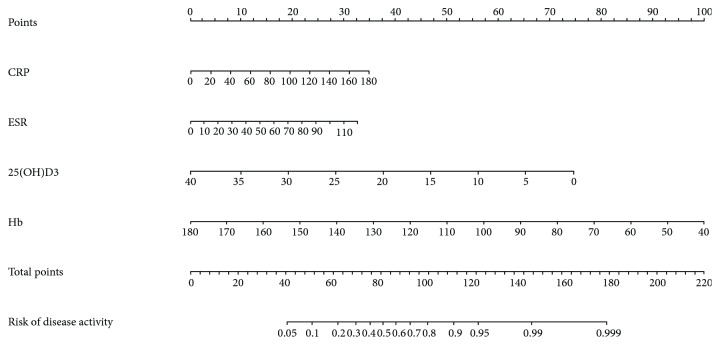
Nomogram for predicting active CD based on 25(OH)D3 and other serum parameters. The c-index of this nomogram was 0.882 with a sensitivity of 73.2% and a specificity of 90.9%.

For better prediction, we set up a new model based on 25(OH)D3 and other serum parameters using Logistic Regression. Since CRP and PLT were highly relevant to ESR with *r*_s_ of 0.594 and 0.530, respectively (*P* < 0.001), they were not included in Logistic Regression. As shown in Table 5, 25(OH)D3 (*P* < 0.001), Hb (*P* < 0.001), and ESR (*P* = 0.013) were related to disease activity with the *B* coefficient of −0.132, −0.046, and 0.022, respectively. To simplify the calculation, we used −(5^∗^25(OH)D3 + 2^∗^Hb) + ESR as a new predictor for disease activity with an AUC of 0.879 (see Table 4 and Figure 1), significantly higher than those of −25(OH)D3 (*P* = 0.0199), CRP (*P* < 0.0001), ESR (*P* < 0.0001), and −Hb (*P* = 0.0396). The optimal threshold value was −272.5 with 72.1% sensitivity and 92.4% specificity.  Figure 2 shows the nomogram using serum parameters for prediction. Internal validation showed a c-index of 0.882 with 73.2% sensitivity and 90.9% specificity.

## 4. Discussion

In this study, we found that 75.1% of CD patients suffered from vitamin D deficiency, as only 3.2% of them had sufficient vitamin D levels. The percentage of patients with vitamin D deficiency was higher than that reported in most previous studies, ranging from 31% to 50%, while the percentage of vitamin D-sufficient patients was much lower than the 35%–73% published in a previous report [16]. However, the results were similar to those of a study on Chinese people, with 67.8% vitamin D deficiency and 3.2% sufficiency in CD patients [25]. Despite no previous study on the differences in vitamin D between Chinese and Caucasian CD patients, one study by Fu et al. pointed out that the prevalence of vitamin D deficiency in South Asian CD patients was higher than that in Caucasian patients [29]. Thus, the higher proportion of vitamin D deficiency in Chinese patients may result from ethnic difference.

Although possible mechanisms of vitamin D deficiency are still unclear, they may include inadequate sun exposure, insufficient dietary intake, absorption dysfunction, impaired conversion of vitamin D activation, increased catabolism, and excretion [16]. Since one of the major sources of vitamin D is absorption by the small intestine, disturbances in the intestinal function may mainly contribute to vitamin D deficiency in CD patients. However, in this study, no significant differences in 25(OH)D3 levels were found either between different disease locations or between with and without previous small intestinal surgery. So malabsorption in the small intestine was also less likely to be the major factor.

Despite vitamin D being a marker of nutrient status in CD patients [17], only few studies have noted the correlation between vitamin D status and BMI in CD patients [30, 31]. Pallav et al.'s study noted a significant association between BMI and vitamin D (*P* = 0.0110), indicating that BMI > 30 kg/m^2^ was a predictor of vitamin D deficiency [31]. Conversely, the other study found no such association [30]. However, these studies were limited by small sample size or absence of subgroup analysis of CD patients. To determine the association between vitamin D and nutrient status, we compared the values of BMI, Hb, and ALB in different groups and performed correlation analysis among them. The results showed that patients with lower vitamin D tend to have lower BMI, Hb, and ALB levels. Moreover, weak but significant positive correlations were found between 25(OH)D3 and BMI, as well as negative correlations between 25(OH)D3 and Hb and ALB. According to these results, we can conclude that vitamin D levels are associated with overall nutrient status in CD patients.

Vitamin D plays an important role in the immune system, especially in patients with CD [32], but previous studies have failed to demonstrate whether the vitamin D status is associated with inflammatory markers like CRP and ESR. Several studies have shown a negative association between 25(OH)D3 and inflammatory markers [33, 34]. A research on a cohort of 201 CD patients in Canada stated that CRP was significantly lower in patients with vitamin D deficiency [33]. Other studies did not reach such an agreement [19, 20, 35, 36]. A study conducted by Garg et al. found a correlation between 25(OH)D3 levels and calprotectin with Pearson's *r* = −0.35 but no correlation with systemic inflammatory markers like CRP and PLT. The authors noted that vitamin D status was associated with only mucosal inflammatory markers but not systemic ones. Because of the conflicting results in previous studies, a study with a larger sample size was needed. In our study, we demonstrated significant negative correlations between 25(OH)D3 and inflammatory biomarkers, which implied that vitamin D may contribute to systemic inflammation in CD and is an efficient biomarker for the estimation of inflammation.

More importantly, our results confirmed a moderate negative association between 25(OH)D3 and disease activity, supporting the study on Chinese patients concerning the correlation, but with a higher absolute value of the correlation coefficient (−0.608 vs. −0.285) [24]. This value was also higher than that reported in the recent meta-analysis on 6 studies [37]. But our result was similar to another study focusing on Chinese patients with a coefficient of 0.582 [26]. Furthermore, we found that the correlation between 25(OH)D3 and CDAI was much higher than that of CRP and ESR. Consequently, the AUC of −25(OH)D3 in ROC analysis for Crohn's disease activity was much higher than the AUCs of CRP and ESR, which indicate that −25(OH)D3 might be a better predictor of disease activity. In addition, we calculated −(5^∗^25(OH)D3 + 2^∗^Hb) + ESR as a new factor to enhance the predictive efficiency of vitamin D for CD activity, which was significantly superior to −25(OH)D3, −Hb, CRP, and ESR, with both higher sensitivity and specificity.

There are some limitations in this study. First, in this single-center retrospective study, most patients inhabited in South China and might not fully represent the overall CD patients in China. Second, the time of follow-up was diverse in different patients. Lack of data on the daily sunlight exposure in different individuals may have caused an inevitable bias in serum levels of 25(OH)D3, as the influence of this factor could not be eliminated in the analysis [38]. Furthermore, some patients with a short follow-up time could be eliminated at the expense of reducing the sample size. Another potential limitation was that the predictive efficacy of markers including −25(OH)D3 and −(5^∗^25(OH)D3 + 2^∗^Hb) + ESR might not be precise, as we used a score of CDAI ≥ 150 to estimate active disease without a gold standard. In addition, we were unable to collect data on intestinal inflammatory biomarkers like fecal calprotectin for demonstrating the association between 25(OH)D3 and intestinal inflammation. We could not use the SESCD score to set up a predictive model either because the number of patients with complete mucosal healing was so limited that it could not meet the need of an effective nomogram. Also, according to the original article presenting the SESCD score, the SESCD score is limited because it is calculated only with the disease behavior under colonoscopy but omits the clinical manifestations, but disease activity is not necessarily reflected on the mucosa [28]. Finally, the CDAI score was correlated with SESCD with a minor *r*_s_ of 0.303, which was consistent with a previous study [39] showing that CDAI is not a reliable measure of the underlying mucosal inflammation. So the SESCD score is not a suitable standard for disease activity in the predictive model. We also lacked the data on the life quality of patients.

In conclusion, despite some inevitable limitations, our study confirms the correlation between vitamin D and the activity of Crohn's disease. We suggest that the correlation between 25(OH)D3 and CDAI might be stronger in Chinese patients. 25(OH)D3 is an efficient marker for estimating systemic inflammation and a better marker for evaluating disease activity than CRP and ESR. Besides, we provide the possibility of using −25(OH)D3 and −(5^∗^25(OH)D3 + 2^∗^Hb) + ESR as predictors for active Crohn's disease, which might be applied in the clinic for distinguishing active diseases. However, these findings need to be verified using further large-scale investigations.

## Figures and Tables

**Table 1:** Demographic and clinical characteristics.

	Group with 25(OH)D3 ≤ 20 ng/ml *N* = 286	Group with 25(OH)D3 > 20 ng/ml *N* = 60	*P*
Gender, *n* (%)			0.005
Male	197 (68.9%)	52 (86.7%)	
Female	89 (31.1%)	8 (13.3%)	
Median age, years (IQR)	26 (20–35)	27 (21–44)	0.212
Median disease duration, years (IQR)	2.0 (0.5–4.4)	1.5 (0.5–5.2)	0.741
Disease location, *n* (%)			0.790
L1	64 (22.4%)	10 (16.7%)	
L2	34 (11.9%)	7 (11.7%)	
L3	169 (59.1%)	39 (65.0%)	
L4	19 (6.4%)	4 (6.7%)	
Disease behavior, *n* (%)			0.308
B1	184 (64.3%)	45 (75.0%)	
B2	69 (24.1%)	9 (15.0%)	
B3	40 (14.0%)	8 (13.3%)	
Perianal lesions, *n* (%)	93 (32.5%)	20 (33.3%)	0.902
Extraintestinal manifestations, *n* (%)	42 (14.7%)	11 (18.3%)	0.476
Previous bowel surgery, *n* (%)	45 (15.7%)	15 (25.0%)	0.085
Previous resection of terminal ileum, *n* (%)	38 (13.3%)	12 (20.0%)	0.179
Previous medications, *n* (%)			
5-Aminosalicylic acid	120 (42.0%)	25 (41.7%)	0.967
Corticosteroids	42 (14.7%)	8 (13.3%)	0.787
Azathioprine/6-mercaptopurine	22 (7.7%)	7 (11.7%)	0.312
Methotrexate	1 (0.3%)	0 (0.0%)	0.646
Thalidomide	8 (2.8%)	2 (3.3%)	0.822
Infliximab	6 (2.1%)	2 (3.3%)	0.563
Sun exposure			0.886
Low (from October to March)	124 (43.4%)	27 (45.0%)	
High (from April to September)	162 (56.6%)	33 (55.0%)	

According to the Montreal classification: L1, terminal ileum; L2, colon; L3, ileocolon; L4, UPG; B1, nonstricturing nonpenetrating; B2, stricturing, B3, penetrating.

**Table 2:** Characteristics of laboratory parameters.

	Group with 25(OH)D3 ≤ 20 ng/ml *N* = 286	Group with 25(OH)D3 > 20 ng/ml *N* = 60	*P*
25(OH)D3, ng/ml (IQR)	11.0 (7.0–14.0)	23.5 (21.0–27.8)	<0.001
ESR, mm/h (IQR)	40.0 (21.0–62.3)	28.0 (13.5–48.8)	0.004
CRP, mg/dl (IQR)	20.6 (5.5–46.9)	11.7 (1.9–24.2)	0.005
WBC, ∗10^9^ (IQR)	7.3 (5.7–9.4)	6.6 (5.4–8.7)	0.089
PLT, ∗10^9^/l (IQR)	341.5 (266.5–413.3)	279.0 (224.0–338.8)	<0.001
Hb, g/l (IQR)	110.0 (93.8–125.0)	127.0 (112.5–139.5)	<0.001
ALB, g/l (mean ± SD)	34.6 (±5.5)	38.0 (±5.1)	<0.001

ESR, erythrocyte sedimentation rate; CRP, C-reactive protein; WBC, white blood cell count; PLT, platelet counts; Hb, hemoglobin; ALB, albumin.

**Table 3:** Correlation of serum 25(OH)D3 levels with CDAI and several parameters.

	*r*_s_	*P*
CDAI score (*N* = 328)	−0.608	<0.001
HBI score (*N* = 18)	−0.516	0.028
SESCD score (*N* = 282)	−0.290	<0.001
BMI(*N* = 326)	0.176	0.001
ESR (*N* = 346)	−0.205	<0.001
CRP (*N* = 346)	−0.219	<0.001
WBC (*N* = 346)	−0.089	0.097
PLT (*N* = 346)	−0.274	<0.001
Hb (*N* = 346)	0.370	<0.001
ALB (*N* = 346)	0.351	<0.001

*r*_s_, correlation coefficient for Spearman correlation; CDAI, Crohn's Disease Activity Index; BMI, body mass index; SESCD, Simplified Endoscopic Score for Crohn's Disease; ESR, erythrocyte sedimentation rate; CRP, C-reactive protein; WBC, white blood cell count; PLT, platelet count; Hb, hemoglobin; ALB, albumin.

**Table 4:** Receiver-operating characteristic analysis for active disease.

	AUC	95% CI of AUC	*P*	Optimal threshold value	Sensitivity	Specificity
−25(OH)D3	0.804	0.753–0.854	<0.001	−12.5	0.611	0.879
CRP	0.693	0.628–0.758	<0.001	27.19	0.468	0.879
ESR	0.713	0.653–0.773	<0.001	46.5	0.454	0.894
−Hb	0.816	0.768–0.864	<0.001	−121.5	0.725	0.803
−(5^∗^25(OH)D3 + 2^∗^Hb) + ESR	0.879	0.841–0.916	<0.001	−518.5	0.682	0.924

AUC, areas under the receiver-operating characteristic curves; ESR, erythrocyte sedimentation rate; CRP, C-reactive protein; Hb, hemoglobin.

**Table 5:** Logistic Regression for disease activity.

Factors	Univariate analysis		Multivariate analysis	
	OR (95% CI)	*P*	OR (95% CI)	*P*
25(OH)D3	0.865 (0.829–0.903)	<0.001	0.876 (0.832–0.923)	<0.001
ESR	1.036 (1.022–1.051)	<0.001	1.022 (1.005–1.040)	0.013
WBC	1.075 (0.968–1.193)	0.177	—	—
Hb	0.934 (0.916–0.952)	<0.001	0.955 (0.934–0.975)	<0.001
ALB	0.826 (0.776–0.879)	<0.001	0.937 (0.863–1.017)	0.121

ESR, erythrocyte sedimentation rate; CRP, C-reactive protein; WBC, white blood cell count; PLT, platelet count; Hb, hemoglobin; ALB, albumin.

## Data Availability

The data were stored in http://ibdcenter.kangzhe.net/ibdcenter/Admin/Login.aspx.
